# The Effect of Ranitidine on Olanzapine-Induced Weight Gain

**DOI:** 10.1155/2013/639391

**Published:** 2013-07-30

**Authors:** Fatemeh Ranjbar, Alireza Ghanepour, Homayoun Sadeghi-Bazargani, Mahbob Asadlo, Amineh Alizadeh

**Affiliations:** ^1^Clinical Psychiatry Research Center, Tabriz University of Medical Sciences, Tabriz 5166614766, Iran; ^2^Neuroscience Research Center, Department of Statistics & Epidemiology, Faculty of Health, Tabriz University of Medical Sciences, Tabriz 5166614711, Iran; ^3^Department of Public Health Sciences, WHO Collaborating Center for Safe Communities, Karolinska Institute, 171 76 Stockholm, Sweden

## Abstract

Induced weight gain is a disturbing side effect of Olanzapine that affects the quality of life in psychotic patients. The aim of this study was to assess the efficacy of Ranitidine in attenuating or preventing Olanzapine-induced weight gain. A parallel 2-arm clinical trial was done on 52 patients with schizophrenia, schizoaffective and schizophreniform disorders who received Olanzapine for the first time. All these were first-episode admitted patients. They were randomly allocated to receive either Ranitidine or placebo. The trend of body mass index (BMI) was compared between groups over 16-week course of treatment. Mean weight was 62.3 (SD: 9.6) kg at baseline. Thirty-three subjects (63.5%) had positive family history of obesity. The average BMI increment was 1.1 for Ranitidine group and 2.4 for the placebo group. The multivariate analysis showed this effect to be independent of sex, family history of obesity, and baseline BMI value. The longitudinal modeling after controlling for baseline values failed to show the whole trend slope to be different. Although the slight change in trend's slope puts forward a hypothesis that combined use of Ranitidine and Olanzapine may attenuate the weight gain long run, this needs to be retested in future larger scale long-term studies. This trial is registered with IRCT.ir 201009112181N5.

## 1. Introduction

New horizons in treatment of psychotic illnesses were explored after the discovery of dual-Serotonin-Dopamine antagonist (second generation antipsychotics) drugs which have been much widely used ever since. Although, compared to the past generations, the second generation antipsychotics have shown different side effects, shortly after consuming drugs as Clozapine and Olanzapine, some undesirable effects ranging from sizeable weight gain and lipid/glucose impairments to serious but rare side effects such as priapism broke out through the course of treatment [1]. The weight gain being a more common problem independent of the dose, develops over time reaching its peak after nine months [2]. The induced weight gain along with its metabolic aftermaths may affect the quality of life [2]. Financial burden of conditions related to weight gain and diabetes has always been of interest [3]. Multiple studies and clinical trials have verified the effect of these drugs in weight gain as large as 30–50 pounds after short-term use of Olanzapine [4]. The weight gain adds to the risk of diabetes mellitus and hyperlipidemia [5, 6]. In order to prevent or diminish the induced weight gain caused by Olanzapine, several types of drugs have been investigated including H2-receptor antagonists such as Nizatidine and Ranitidine [7, 8] as well as selective serotonin reuptake inhibitors such as Fluoxetine, topiramate, reboxetine-betahistine, aripiprazole, and Amantadine [9, 10]. Ranitidine has a fairly reasonable price and high compliance and can be used as alternative in this regard; nevertheless available knowledge about its effect on Olanzapine-induced weight gain is quite scarce.

The aim of this study was to assess the efficacy of Ranitidine in attenuating or preventing Olanzapine-induced weight gain.

## 2. Materials and Methods 

**Figure 1:**
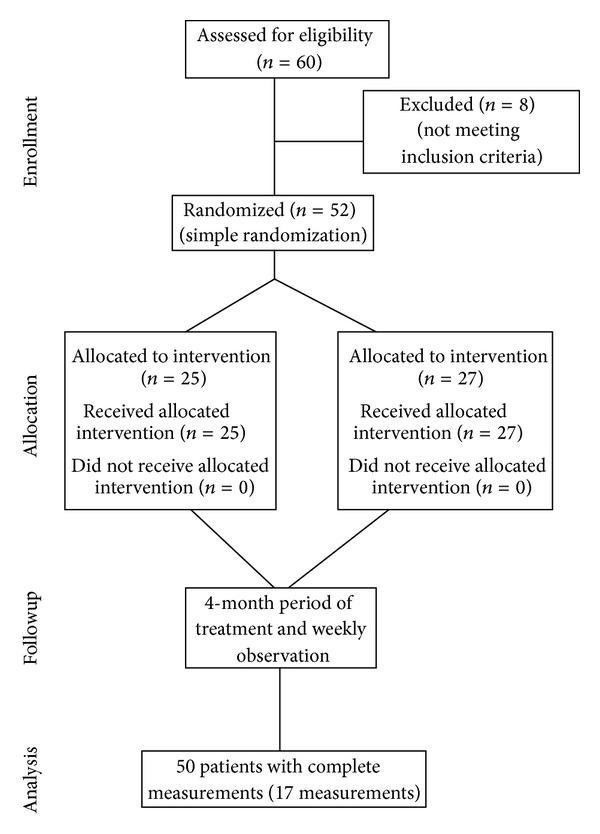
CONSORT diagram showing the flow of participants through each stage of study to compare effect of Ranitidine with placebo on Olanzapine-induced weight gain.

*Design and Participants.* A randomized clinical trial was conducted in Razi University Hospital in Tabriz, Iran, in 2009. The study participants included patients admitted to psychiatry ward of Razi University Hospital with a diagnosis of schizophrenia schizoaffective and schizophreniform disorders according to DSM-IV criteria and were planned to be treated with Olanzapine. A parallel two-arm study design was applied. Fifty-two out of 60 patients screened for eligibility were enrolled. The CONSORT flowchart provides further details as in Figure 1.

The inclusion criteria other than the DSM-IV diagnosis were as follows:

1. Olanzapine consumption,
2. planned hospitalization for more than 16 weeks while receiving Olanzapine,
3. the official informed consent of the patients' authorized guardian.

The exclusion criteria were as follows:

1. presence of comorbid physical illnesses,
2. simultaneous use of drugs that might affect weight,
3. following specific diets other than those provided by the ward as a routine.

## 3. Interventions and Outcomes

*Treatment Protocol.* For test group the treatment was started with 600 mg/day (300 mg BID) of Ranitidine as tablets prescribed for oral consumption. Treatment continued for 16 weeks. The patients in control group were given a placebo tablet with identical shape, taste, size, odor, and color of Ranitidine. The drug tablets were produced by Sobhan Pharmaceutical Company.

Placebo tablets were made by Industrial Department of the Faculty of Pharmacy, Tabriz University of Medical Sciences. All the trial patients received Ranitidine as well as Olanzapine, and the control patients received placebo with Olanzapine for 16 weeks. 

The primary outcome of the study was body mass index (BMI) trend assessed based on weekly measurement of weight and height. Measurements were done using calibrated and standard instruments. Height was measured with participants standing without shoes using a standard metal ruler. Weight was measured also by calibrated digital scales while light clothes were worn. 

### 3.1. Randomization and Blinding

The eligible subjects were randomly allocated into two groups as shown in Figure 1. The random sequence was generated using simple randomization method [11]. To conceal the allocation from the researchers, the ward nurse matched the random sequence with admission registration order and announced the allocation group code. The medical practitioners, patients, and the statistical epidemiologist who did the statistical analysis were kept blind of the medication type ensuring a triple blinding procedure. 

### 3.2. Statistical Analysis

Data were analyzed using SAS 9.1 statistical software package. Both descriptive and analytical statistical methods were used. The drug effects were compared on the basis of a one-sided equality hypothesis comparing BMI trend over 16 weeks of treatment [12]. With regard to the correlated multiple measurements of outcome, longitudinal data analysis methods were applied. Considering the number of measurements and missed times, covariance modeling was used as an overall analysis strategy in lieu of average modeling. Compound Symmetry, TOEPLITZ, and Autoregressive models were the three covariance modeling techniques used. Based on AIC and model specifications and the assumptions, TOEPLITZ was selected as the ultimate model. As the secondary outcome of the study, mean before-after change in BMI was compared between the groups using independent samples *t-*test. The level of significance was set at 0.05 in interpreting the results of statistical analysis.

### 3.3. Ethics

The study protocol and primary outcome of the study did not change over the course of research. Study protocol was approved by the responsible committee of ethics at Tabriz University of Medical Sciences. 

## 4. Results 

Men comprised 63.5% of the participants. Mean (standard deviation) of age of the participants was 38.1 (11), and the mean (standard deviation) of the height was 165 (9.6) cm. Mean weight was 62.3 (SD: 9.6) kg at baseline. Thirty-three subjects (63.5%) had positive family history of obesity. These measurements are compared between the groups in Table 1. 

BMI variations through the first and the 17th measurements with respect to gender and family history were as presented in Table 2. As could be found in this table, mean BMI change through the course of treatment was higher in women than in men. In addition, those who had positive family history for obesity had higher increment in BMI when compared with those lacking such a family history.

**Figure 2:**
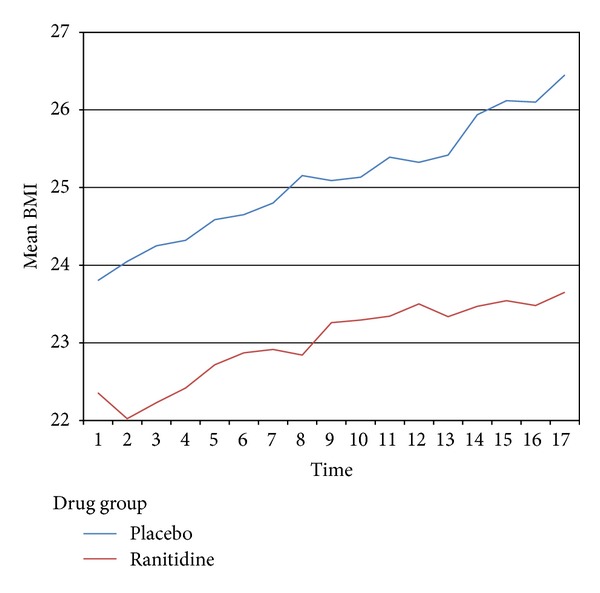
BMI trends comparing placebo and ranitidine groups over the 16-week course of treatment with Olanzapine. *x*-axis: measurement sessions from baseline (1) to the end of 16th week (17). *y*-axis: mean body mass index (BMI).

BMI trends were compared both for placebo and trial groups as could be found in Figure 2. As could be seen in this graph the BMI increased with equal slope in both groups up to the eighth week, whereas, the BMI trend slope decreased after this time point in ranitidine group. 

Mean baseline BMI was 0.2 kg/m^2^ lower in ranitidine group without statistical significance. Mean BMI in Ranitidine group was 2.8 kg/m^2^ lower than the placebo group 16 weeks after the onset of the treatment, and the observed difference happened to be statistically significant (*P* < 0.01, *t* = 2.5). The average BMI increment, from the baseline to the end of the study course after 16 weeks, was 1.1 for Ranitidine group and 2.4 for the placebo group. BMI change in Ranitidine group was 1.3 kg/m^2^ less than the placebo group (*P* < 0.05, *t* = 2). Thus the results indicated that the BMI had increased in both groups through the first 16 weeks of the study but the weight gain was slightly attenuated among patients receiving ranitidine. 

After controlling for baseline values and other cofactors, the longitudinal data modeling for slope comparison failed to show the whole trend slope to be different for the two groups. 

The longitudinal model showed also that a positive family history of obesity increased the likelihood of Olanzapine-induced weight gain. 

## 5. Discussion 

Treatment with Olanzapine and Clozapine is shown to cause weight gain that in turn may increase the risk of type 2 diabetes by affecting the glucose metabolism. Olanzapine is an antihistamine and strong antagonist of the muscarinic M3 receptor, which may explain its diabetes side effects [13, 14]. 

Also it has been shown that these drugs increase triglyceride, hence the risk of coronary artery disease. This issue should be taken into consideration when prescribing such medications, or, preferably, certain solutions should be sought in order to prevent or reduce these side effects [15, 16]. The histamine H2 receptor is shown to be one of the possible mediators of feeding behavior and weight regulation [17], thus supporting the plausibility of using H2 antagonists for controlling the induced weight gain. 

In the present study, BMI and weight increased over the 16 weeks of treatment both in placebo and Ranitidine groups that were treated by Olanzapine. In most studies, with or without preventives, Olanzapine has resulted in an increased weight gain [7, 8, 18–24]. However, it has also been found that Olanzapine may not lead to weight gain in all patients and may even be associated with weight loss in about 10 percent of the consumers [25]. The effect of weight gain has been proved to reach the plateau level within 52 weeks [9]. 

Assessing the effect of ranitidine on weight gain trend in the present study, the longitudinal data modeling for slope comparison failed to show the whole trend slope to be different. Although during the weeks in second half of the study period the slopes were found to be slightly different between the groups, regardless of statistical significance, it was not the primary objective of the study to compare just the second part of the trends. However, this may suggest a hypothesis that ranitidine may in the long run attenuate the weight gain induced by Olanzapine. The multivariate analysis showed this slope difference to be independent of sex, family history of obesity, and baseline BMI value.

In order to prevent or minimize Olanzapine-induced weight gain, researchers have investigated various techniques using different dosage or galenical forms and administration routes of the drug have been assessed as possible solutions to reduce the weight gain. This has been considered successful in some studies [26–28]. As the tolerance of glucose is affected by Olanzapine, some researches have recommended of use Metformin. Nevertheless, controversial results have been reached in finding it effective [24] or without efficacy [19, 20]. 

Since H2-receptor antagonists have fewer side effects, fairly reasonable price, and better availability, they could be preferable options to be used in minimizing the weight gain induced by second generation antipsychotics. Nizatidine has gained more attention in this regard and some studies have found it to be effective in alleviating or preventing weight gain induced by Olanzapine [7, 16, 21, 23, 29]. Nevertheless, Nizatidine has also been reported as noneffective in alleviating or preventing Olanzapine-induced weight gain. Available knowledge regarding other H2-antagonists like famotidine and ranitidine is scarce and inconclusive. 

Famotidine (40 mg/day) has been compared with placebo in a previous randomized clinical trial on 14 first-episode schizophrenia patients for 6 weeks to assess its efficacy when given as an adjunct to Olanzapine (10 mg/day) [22]. The study failed to find famotidine as effective in this regard. However, the results of the famotidine study seem to be inconclusive both due to very small sample size and short course of study. In our study also the authors found that Ranitidine was not different from placebo over the first eight weeks of treatment. As our study continued for 16 weeks, it was possible to detect the delayed efficacy of ranitidine in attenuating the weight gain induced by Olanzapine. However, the researchers are not even convinced that 16 weeks of study to investigate the effects of ranitidine are sufficient and recommend future research with longer period of study.

The authors were able to retrieve only one similar study about ranitidine. The study was published in Spanish and we were not able to explore the details of study design. However, they had found ranitidine to be effective in attenuating the weight gain caused by Olanzapine. They found that Ranitidine as an adjunct treatment to Olanzapine corrected weight gain in 59.6% of patients and mean weight gain was lower in those patients receiving adjunct Ranitidine [8].

The multivariate model in our study found the family history of obesity as well as baseline BMI status of the patients as predictors of weight gain induced by Olanzapine. This is partly consistent with previous research. To predict the rate of Olanzapine-induced weight gain, genetic factors, leptin level, baseline BMI, and gender have been proposed in previous research [29–31]. 

## 6. Conclusion 

Concomitant use of Ranitidine and Olanzapine treatment does not prevent Olanzapine-induced weight gain during the first two months of the study and does not appear to cause different weight gain trend slopes over the first four months of the treatment. Although the slight change in trend slope puts forward a hypothesis that the combined use of Ranitidine and Olanzapine may attenuate the weight gain in the long term, this needs to be tested in larger scale long-term studies. 

## Figures and Tables

**Table 1:** Baseline comparisons between the trial groups.

	Placebo	Ranitidine
Age/years: mean (SD)	37.7 (11)	38.5 (11.2)
Weight/kg: mean (SD)	63.2 (8.1)	61.4 (11.5)
BMI: mean (SD)	23.8 (3.5)	22.4 (3.1)
Family history of obesity: number (%)	Positive: 11 (40.7) Negative: 16 (59.3)	Positive: 8 (32) Negative: 17 (68)

**Table 2:** Descriptive statistics of BMI before-after change compared for gender and family history of obesity.

	Mean change in BMI	SD (change in BMI)	Median (change in BMI)	Min. (change in BMI)	Max. (change in BMI)
BMI change with respect to sex					
Male	1.2	2.4	0.9	−3	6.4
Female	2.8	1.9	2.7	−0.4	7.4
BMI change with respect to family history					
Positive	2.4	2	1.6	−0.3	7.4
Negative	1.4	2.5	1	−3	6.4
Overall BMI change through the course of treatment	1.8	2.3	1.6	−3	7.4

Positive values mean increment in BMI.

## References

[1] Hosseini SH, Polonowita AK (2009). Priapism associated with olanzapine. *Pakistan Journal of Biological Sciences*.

[2] Sadock B, Sadock V (2007). *Synopsis of Psychiatry*.

[3] Ghaffari S, Hashemi SE, Atabaki H (2012). The national financial burden of hospitalization of diabetes in Iran. *Journal of Clinical Research & Governance*.

[4] Gupta S, Droney T, Al-Samarrai S, Keller P, Frank B (1999). Olanzapine: weight gain and therapeutic efficacy. *Journal of Clinical Psychopharmacology*.

[5] Goff DC, Evins AE (1998). Negative symptoms in Schizophrenia: neurobiological models and treatment response. *Harvard Review of Psychiatry*.

[6] Siris SG, van Kammen DP, Docherty JP (1978). Use of antidepressant drugs in schizophrenia. *Archives of General Psychiatry*.

[7] Cavazzoni P, Tanaka Y, Roychowdhury SM, Breier A, Allison DB (2003). Nizatidine for prevention of weight gain with olanzapine: a double-blind placebo-controlled trial. *European Neuropsychopharmacology*.

[8] López-Mato A, Rovner J, Illa G, Vieitez A, Boullosa O (2003). Randomized, open label study on the use of ranitidine at different doses for the management of weight gain associated with olanzapine administration. *Vertex*.

[9] Andersen SW, Clemow DB, Corya SA (2005). Long-term weight gain in patients treated with open-label olanzapine in combination with fluoxetine for major depressive disorder. *Journal of Clinical Psychiatry*.

[10] Davis KL, Kahn RS, Ko G, Davidson M (1991). Dopamine in schizophrenia: a review and reconceptualization. *American Journal of Psychiatry*.

[11] Hajebrahimi S, Mostafaie A, Sadeghi-Bazargani H (2011). Evidence for the future—designing a clinical trial. *Indian Journal of Urology*.

[12] Sadeghi-Bazargani H, Sedghipour M (2012). Setting the objectives and hypotheses in randomized clinical trials: notices for clinicians and pharmacologists. *International Journal of Pharmacology*.

[13] Johnson DE, Yamazaki H, Ward KM (2005). Inhibitory effects of antipsychotics on carbachol-enhanced insulin secretion from perifused rat islets: role of muscarinic antagonism in antipsychotic-induced diabetes and hyperglycemia. *Diabetes*.

[14] Kast RE, Altschuler EL (2006). Current drugs available now for interleukin-6 suppression as treatment adjunct in glioblastoma: anakinra, aprepitant, mirtazapine and olanzapine. *International Journal of Cancer Research*.

[15] Eder-Ischia U, Ebenbichler C, Fleischhacker WW (2005). Olanzapine-induced weight gain and disturbances of lipid and glucose metabolism. *Essential Psychopharmacology*.

[16] Hester EK, Thrower MR (2005). Current options in the management of olanzapine-associated weight gain. *Annals of Pharmacotherapy*.

[17] Doi T, Sakata T, Yoshimatsu H (1994). Hypothalamic neuronal histamine regulates feeding circadian rhythm in rats. *Brain Research*.

[18] Assunção SSM, Ruschel SI, Rosa LDCR (2006). Weight gain management in patients with schizophrenia during treatment with olanzapine in association with nizatidine. *Revista Brasileira de Psiquiatria*.

[19] Baptista T, Martínez J, Lacruz A (2006). Metformin for prevention of weight gain and insulin resistance with olanzapine: a double-blind placebo-controlled trial. *Canadian Journal of Psychiatry*.

[20] Baptista T, Uzcátegui E, Rangel N (2008). Metformin plus sibutramine for olanzapine-associated weight gain and metabolic dysfunction in schizophrenia: a 12-week double-blind, placebo-controlled pilot study. *Psychiatry Research*.

[21] Pae C-U, Kim J-J, Lee K-U (2003). Effect of nizatidine on olanzapine-associated weight gain in schizophrenic patients in Korea: a pilot study. *Human Psychopharmacology*.

[22] Poyurovsky M, Tal V, Maayan R, Gil-Ad I, Fuchs C, Weizman A (2004). The effect of famotidine addition on olanzapine-induced weight gain in first-episode schizophrenia patients: a double-blind placebo-controlled pilot study. *European Neuropsychopharmacology*.

[23] Sacchetti E, Guarneri L, Bravi D (2000). H_2_ antagonist nizatidine may control olanzapine-associated weight gain in schizophrenic patients. *Biological Psychiatry*.

[24] Wu R-R, Zhao J-P, Guo X-F (2008). Metformin addition attenuates olanzapine-induced weight gain in drug-naive first-episode schizophrenia patients: a double-blind, placebo-controlled study. *American Journal of Psychiatry*.

[25] Bushe C, Sniadecki J, Bradley AJ, Hoffmann VP (2010). Comparison of metabolic and prolactin variables from a six-month randomised trial of olanzapine and quetiapine in schizophrenia. *Journal of Psychopharmacology*.

[26] Arranz B, San L, Dueñas RM (2007). Lower weight gain with the orally disintegrating olanzapine than with standard tablets in first-episode never treated psychotic patients. *Human Psychopharmacology*.

[27] Karagianis J, Hoffmann VP, Arranz B (2008). Orally disintegrating olanzapine and potential differences in treatment-emergent weight gain. *Human Psychopharmacology*.

[28] van der Zwaal EM, Luijendijk MCM, Adan RAH, la Fleur SE (2008). Olanzapine-induced weight gain: chronic infusion using osmotic minipumps does not result in stable plasma levels due to degradation of olanzapine in solution. *European Journal of Pharmacology*.

[29] Atmaca M, Kuloglu M, Tezcan E, Ustundag B (2003). Nizatidine treatment and its relationship with leptin levels in patients with olanzapine-induced weight gain. *Human Psychopharmacology*.

[30] Atmaca M, Tezcan E, Ustundag B (2007). Plasma nitric oxide and leptin values in patients with olanzapine-induced weight gain. *Journal of Psychiatric Research*.

[31] Baptista T, Beaulieu S (2001). Body weight gain, insulin, and leptin in olanzapine-treated patients. *Journal of Clinical Psychiatry*.

